# Epigenetically active chromatin in neonatal iWAT reveals GABPα as a potential regulator of beige adipogenesis

**DOI:** 10.3389/fendo.2024.1385811

**Published:** 2024-05-03

**Authors:** Raja Gopal Reddy Mooli, Bokai Zhu, Saifur R. Khan, Veerababu Nagati, Kulandaimanuvel Antony Michealraj, Michael J. Jurczak, Sadeesh K. Ramakrishnan

**Affiliations:** ^1^ Division of Endocrinology and Metabolism, University of Pittsburgh, Pittsburgh, PA, United States; ^2^ Pittsburgh Liver Research Centre, University of Pittsburgh, Pittsburgh, PA, United States; ^3^ Aging Institute of University of Pittsburgh Medical Center (UPMC), University of Pittsburgh, Pittsburgh, PA, United States; ^4^ Division of Cardiology, University of Pittsburgh, Pittsburgh, PA, United States; ^5^ Vascular Medicine Institute, University of Pittsburgh, Pittsburgh, PA, United States; ^6^ Pittsburgh VA Medical Centre, Pittsburgh, PA, United States; ^7^ Center for Immunometabolism, University of Pittsburgh, Pittsburgh, PA, United States

**Keywords:** beige adipocyte, neonatal adipose tissue, epigenetic modification, GABPA, subcutaneous adipose tissue

## Abstract

**Background:**

Thermogenic beige adipocytes, which dissipate energy as heat, are found in neonates and adults. Recent studies show that neonatal beige adipocytes are highly plastic and contribute to >50% of beige adipocytes in adults. Neonatal beige adipocytes are distinct from recruited beige adipocytes in that they develop independently of temperature and sympathetic innervation through poorly defined mechanisms.

**Methods:**

We characterized the neonatal beige adipocytes in the inguinal white adipose tissue (iWAT) of C57BL6 postnatal day 3 and 20 mice (P3 and P20) by imaging, genome-wide RNA-seq analysis, ChIP-seq analysis, qRT-PCR validation, and biochemical assays.

**Results:**

We found an increase in acetylated histone 3 lysine 27 (H3K27ac) on the promoter and enhancer regions of beige-specific gene UCP1 in iWAT of P20 mice. Furthermore, H3K27ac ChIP-seq analysis in the iWAT of P3 and P20 mice revealed strong H3K27ac signals at beige adipocyte-associated genes in the iWAT of P20 mice. The integration of H3K27ac ChIP-seq and RNA-seq analysis in the iWAT of P20 mice reveal epigenetically active signatures of beige adipocytes, including oxidative phosphorylation and mitochondrial metabolism. We identify the enrichment of GA-binding protein alpha (GABPα) binding regions in the epigenetically active chromatin regions of the P20 iWAT, particularly on beige genes, and demonstrate that GABPα is required for beige adipocyte differentiation. Moreover, transcriptomic analysis and glucose oxidation assays revealed increased glycolytic activity in the neonatal iWAT from P20.

**Conclusions:**

Our findings demonstrate that epigenetic mechanisms regulate the development of peri-weaning beige adipocytes via GABPα. Further studies to better understand the upstream mechanisms that regulate epigenetic activation of GABPα and characterization of the metabolic identity of neonatal beige adipocytes will help us harness their therapeutic potential in metabolic diseases.

## Introduction

Adipose tissue is well-known for its role in fat storage; however, it can also suppress weight gain, improve whole-body metabolic homeostasis, and defend against fasting, exercise, and cold exposure (1–3). These distinct functions of adipose tissue are performed by brown adipose tissue (BAT) localized in distinct anatomical sites, such as the interscapular, cervical, perirenal, and axillary depots (4). BAT comprises brown adipocytes characterized by multilocular lipid droplets, abundant mitochondrial content, and uncoupling protein 1 (UCP1) expression. UCP1, also known as “thermogenin”, is a mitochondria-specific protein that uncouples cellular respiration and mitochondrial ATP synthesis to dissipate energy in the form of heat (5). Brown adipocytes also emerge in inguinal white adipose tissue (iWAT), known as beige fat, through a process referred to as browning or beiging (6). Similar to brown adipocytes, beige adipocytes possess multilocular lipid droplets, high mitochondrial content, and UCP1 expression (5, 7). In mice and adult humans, beige fat is induced by external stimuli, such as cold (8), exercise (9), calorie restriction (10, 11), and β-adrenergic receptor agonists (12). At the molecular level, beiging of white adipose tissue was regulated by various transcriptional factors (5, 13), miRNAs (14), epigenetic factors such as DNA methylases (15), and histone-modifying enzymes (16).

Seminal studies have identified beige adipocytes in neonatal iWAT as early as postnatal day 14 (P14) and peaks at approximately P21–25 (17, 18). Unlike cold-induced beige, neonatal beige adipocytes develop from myogenic precursors in a temperature- and sympathetic innervation-independent manner (17, 19). Indeed, factors secreted from the neonatal beige adipocytes drive sympathetic innervation of iWAT (20). Although neonatal beige adipocytes regress in adulthood, tracing experiments show that ~40%–50% of inducible beige adipocytes are formed from the rejuvenated neonatal beige adipocytes (21, 22), suggesting that neonatal beige plays a crucial role in thermogenesis. Many studies have focused on cold-induced recruitment of beige adipocytes and identified the transcription factors and signaling mechanisms, mostly by modulating sympathetic nerves, that densely innervate in iWAT (7, 13, 23). However, the transcriptional regulators or signaling mechanisms that control neonatal beige adipogenesis remain largely unknown.

We report that the induction of neonatal beige in the iWAT of P20 mice is associated with higher levels of histone H3 acetylation on lysine 27 (H3K27ac), a marker for active enhancers and promoters (24). ChIP analysis showed higher H3K27ac levels in the promoter and enhancer regions of UCP1, a classical thermogenic marker for brown and beige fat (25). Furthermore, H3K27ac ChIP-seq revealed epigenetically active enhancer regions of brown/beige genes but not white genes, suggesting epigenetic activation of beige genes. Integrating the epigenetic and transcriptomic analysis identified the enrichment of genes/pathways involved in the thermogenesis program, including oxidative phosphorylation and mitochondrial metabolism. We then performed motif enrichment analysis using H3K27ac peaks and discovered a higher representation of the binding site for the transcription factor GA binding protein α (GABPα), which has recently been shown to induce glycolytic beige fat (26). Consistently, neonatal beige adipocytes express higher levels of glycolytic genes and oxidize glucose, and knockdown *Gabpα* in SVF cells abrogates beige adipocyte differentiation *in vitro*. Collectively, we report that epigenetic mechanisms via GABPα induce neonatal beige fat that is glycolytic.

## Materials and methods

### Animals

All animal studies were conducted under an approved Institutional Animal Care and Use Committee (IACUC) protocol at the University of Pittsburgh. All the mice are in a C57BL6 background and housed in the mouse vivarium at ambient temperatures. Mice were housed individually in a specific pathogen-free facility with 12-h day and night cycles and provided *ad libitum* access to a normal chow diet provided by the DLAR. Both male and female mice of various ages were used in the study.

### Cell culture, siRNA transfection, and differentiation

Mouse inguinal stromal vascular fraction (SVF) cell lines purchased from Kerafast, Inc. (Boston, MA) were cultured in Dulbecco’s modified Eagle’s medium/F12 (DMEM/F12) containing 10% fetal bovine serum (FBS) and 1% penicillin/streptomycin (27). For knockdown experiments, 60%–70% confluent SVF cells were transfected with 30 pmol scrambled or pre-designed siRNA against *Gabpα* (Cat # AM16708, ID: 157638; Ambion by Life Technologies, Thermofisher Scientific; sense 5′-GCAUUGUGGAACAACCUAtt-3′ and antisense 5′-UAGGUUUGUUCCACAAUGCtt-3′) using Lipofectamine RNAiMAX transfection reagent (Thermofisher Scientific) following the manufacturer’s instructions. Twenty-four hours post-transfection, SVF cells were differentiated with DMEM/F12 media containing 10% FBS, 0.5 mM isobutylmethylxanthine, 125 nM indomethacin, 1 μM dexamethasone, 850 nM insulin, 1 nM T_3_, and 1 μM rosiglitazone for 48 h and then switched to the maintenance media (DMEM/F12 with 10% FBS, 850 nM insulin, 1 nM T_3_, and 1 μM rosiglitazone). Maintenance media was refreshed every 48 h for 6 days.

### Oil red O staining

Oil Red O staining was performed in differentiated mouse SVF cells as described earlier (28). Briefly, cells were washed with 1× PBS and fixed with 10% PBS-buffered formalin at room temperature for 30 min, and then washed with ddH_2_O. The cells were incubated with 60% isopropyl alcohol for 5 min and stained with Oil Red O (Sigma-Aldrich) for 30–45 min. The cells were washed several times with ddH_2_O and counterstained with hematoxylin for 15 s. The images were captured using an EVOS microscope (Olympus, Tokyo, Japan).

### Western blotting

For preparation of whole cell extracts, frozen iWAT tissue samples were homogenized in a RETSCH MM 400 Mixer Mill (Fisher Scientific, Hampton, NH) for 90 s at 30 Hz using RIPA buffer (50 mM Tris-Cl, pH 7.5, 150 mM NaCl, 1% Nonidet P-40 substitute, 0.1% SDS, 0.5% sodium deoxycholate, and 1 mM EDTA), with protease inhibitor cocktail and phosphatase inhibitor (Sigma Aldrich, St Louis, MO). After quantifying protein concentration with a Pierce BCA Protein Assay Kit (Bio-Rad, Hercules, CA), 10–20 µg of protein was separated by SDS-PAGE and transferred to a 0.45-µm nitrocellulose membrane (Bio-Rad). The membrane was blocked for 30 min using 5% nonfat dry milk in Tris-buffered saline (TBS) containing 0.1% Tween-20, and probed with antibodies (details were provided in **Table 1**) overnight at 4°C. After washing with TBST, blots were probed with secondary antibodies (Anti-mouse IgG DyLight 680 or anti-rabbit DyLight 800, Cell Signaling Technology, Boston, MA) and visualized using the Odyssey CLx Imaging System (LI-COR, Lincoln, NE).

**Table 1:** Antibody details.

#	Name	Catalog number	Source
1	Actin	66009-1-Ig	Proteintech
2	H3K27ac	#8173	Cell Signaling Technology
4	H3K9ac	#9649	Cell Signaling Technology
5	GABPα	Sc-28312	Santa Cruz Biotechnology
6	PKM1/2	#3190	Cell Signaling Technology
7	HK2	#2867	Cell Signaling Technology
8	LDHA	#3582	Cell Signaling Technology
9	PGC-1α	ab54481	Abcam
10	SDHA	#5839	Cell Signaling Technology
11	Tom20	#42406S	Cell Signaling Technology
12	UCP1	PA1-24894	Invitrogen
13	Histone H3	#9715	Cell Signaling Technology
14	FABP4	#2120	Cell Signaling Technology
15	GAPDH	Sc-32233	Santa Cruz Biotechnology
16	PPARg	#2430	Cell Signaling Technology

### Immunohistology

Freshly isolated iWAT was fixed in 10% PBS-buffered formalin, dehydrated, and embedded in paraffin for sectioning. Five-micron sections were de-paraffinized, and the antigen was retrieved using 10 mM sodium citrate buffer containing 0.05% Tween20, pH 6.0. After blocking the endogenous peroxidases and non-binding sites with superblock (ScyTek Laboratories Inc., Logan, UT), the sections were incubated with primary antibodies (UCP1, 1:250; TOM20, 1:250, Cell Signaling Technology, and GABPα, 1:100, Santa Cruz Biotechnology) overnight at 4°C. After several washes, the slides were incubated with Dky Rabbit IgG Biotin secondary antibody (1:500, EMD Millipore, Burlington, MA) and developed using the DAB peroxidase substrate kit (Vector Laboratories, Burlingame, CA). The color development time was optimized by monitoring the signal under a microscope. The nuclei were stained with hematoxylin, the slides were mounted using a Permount mounting medium (Fischer Scientific), and the images were captured using an EVOS microscope.

### RNA isolation and qRT-PCR

iWAT samples were snap-frozen in liquid nitrogen and homogenized in Trizol (Invitrogen, Waltham, MA) using RETSCH MM 400 Mixer Mill (Fisher Scientific) for 60 s at 30 Hz. RNA concentration was measured with a Nanodrop 2000 spectrophotometer (Fisher Scientific). RNA (1 µg) was reverse-transcribed using Mu-MLV reverse transcriptase (Promega, Madison, WI). The relative expression of genes was assessed using 2× SYBR Green master mix (APExBIO, Houston, TX) in QuantStudio 3 Station qPCR machine (Applied Biosystems, Foster City, CA). The average cycle threshold value (CT) of the reference mRNA (actin) was subtracted from the average CT of the target mRNA to yield the ΔCT value. The primer sequences are provided in **Table 2**.

**Table 2:** Primer sequences for qRT-PCR and ChIP-PCR.

No.	Gene	Forward (5′-3′)	Reverse (5′-3′)
1	*Ldha*	ACTTGGCGCTCTACT TGCTG	AGGGTTGCCATCTTGGAC TTT
2	*Pkm2*	GCCGCCTGGACATT GACTC	CCATGAGAGAAATTCAGC CGAG
3	*Pdha*	GAAATGTGACCTTC ATCGGCT	TGATCCGCCTTTAGCTCC ATC
4	*Pfkp*	CGCCTATCCGAAGT ACCTGGA	CCCCGTGTAGATTCCCATGC
5	*Pdhb*	TGGCAGCAGGTG TCTCTGTA	TATCTTCCATGGGGGCATT
6	*Gapdh*	TGAAGGTCGGTGTG AACG	CCATTCTCGGCCTTGACT
7	*Glut1*	CAAGTCTGCATTGC CCATGAT	CCAGCTGGGAATCGTCGTT
8	*Gabpa*	AGCGCATCTCGTTG AAGAAG	TCCTGCTCTTTTCTGTAG CCT
9	*Ucp1*	ACTGCCACACCTCC AGTCATT	CTTTGCCTCACTCAGGAT TGG
10	*β-Actin*	TATTGGCAACGAGC GGTTCC	GGCATAGAGGTCTTTACG GATGT
11	*Cidea*	AGGGACAGAAATGG ACACCG	GCAGATTCCTTAACACGG CCT
12	*Dio2*	AATTATGCCTCGGA GAAGACCG	GGCAGTTGCCTAGTGAAA GGT
13	*Nd2*	GCCTGGAATTCAGC CTACTAGC	GGCTGTTGCTTGTGTGAC GA
14	*Elovl3*	CTCTTTCTTCTCAGC AAGGT	TGTACATGACAGAATGG ACG
15	*Prdm16*	CAGCACGGTGAAGC CATTC	GCGTGCATCCGCTTGTG
16	*Il-4*	AGATGGATGTGCCA AACGTCCTCA	AATATGCGAAGCACCTTGG AAGCC
17	*F4/80*	CTTTGGCTATGGGC TTCCAGTC	GCAAGGAGGACAGAGTTTA TCGTG
18	*Arg*	ACCTGGCCTTTGTT GATGTCCCTA	AGAGATGCTTCCAACTGCC AGACT
19	*Th*	GGCTTCTCTGACCA GGCGTAT	TGCTTGTATTGGAAGGCAA TCTC
17	*Ucp1* enhancer	CTCCTCTACAGCGT CACAGAGG	AGTCTGAGGAAAGGGTTGA
18	*Ucp1* proximal	CCCACTAGCAGCTC TTTGGA	CTGTGGAGCTCAAAGGT

### *Ex vivo* 3-[^3^H]-glucose quantification of glycolysis

Freshly isolated iWAT tissue samples were weighed (~30 mg) and transferred to a six-well culture plate with 1 mL of DMEM media and minced into small pieces. The tissue samples were incubated with control media (DMEM, 200 mM 2-deoxyglucose, and 0.2 μCi 3-[^3^H]-glucose) and experimental media (DMEM and 0.2 μCi 3-[^3^H]-glucose) for 30 min. The media with the samples were spun at 10,000*g* for 10 min at 4°C. The supernatant (600 μL) was collected and aliquoted into two scintillation vials (300 μL each), one as “dry” and the other as “non-dry”. The non-dry samples were mixed with a liquid scintillation cocktail immediately and counted, whereas dry samples were dried in a heated vacuum overnight, reconstituted in 4.5 mL of scintillation media, and counted the next day. The radioactive counts of the dry sample were subtracted from non-dry counts, where the difference represents ^3^H_2_O produced during glycolysis.

### Nuclear isolation and ChIP-qPCR analysis

Nuclear fractions from iWAT were isolated as previously described (16). Frozen iWAT samples were minced into small pieces and homogenized using a dounce (7 mL) in nucleus preparation buffer [NPB; 10 mM HEPES (pH 7.5), 1.5 mM MgCl_2_, 10 mM KCl, 250 mM sucrose, 0.1% NP-40, and 0.2 mM DTT]. Homogenates were filtered through 100-μm cell strainers and cross-linked with 1% paraformaldehyde (PFA) at room temperature for 4 min while rotating and quenched by 125 mM glycine for 10 min. The homogenates were centrifuged at 1,000*g* for 10 min and the nuclei-containing pellets were washed once in NPB and spun down at 1,000*g* for 10 min. The nuclei pellet was resuspended in nuclei lysis buffer [NLB, 10 mM Tris (pH 8), 1 mM EDTA, 0.1% SDS] and sheared using a Qsonica E220 (Newtown, CT) with the following parameters: amplitude 70%, cycles on for 15 s, cycles off for 45 s, with a total time of 45 min. The sheared chromatin was centrifuged at 13,000 rpm for 10 min at 4°C to remove debris and quantified using nanodrop. Sheared chromatin (2.5 μg) was diluted in ChIP dilution buffer [CDB, 16.7 mM Tris (pH 8), 1.2 mM EDTA, 167 mM NaCl, 1.1% Triton X-100, and 0.01% SDS] and incubated with antibodies H3K27ac (1 μg/mL; Active Motif, 39133) and GABPα (2 μg/mL; Santa Cruz Biotechnology, sc-28312X) or normal rabbit IgG overnight while rotating at 4°C. Protein A/G dynabeads were washed in PBS/1% BSA and added to the ChIP samples. After rotating for 2 h at 4°C, the samples were processed as per the ChIP-IT Express kit instructions (Active Motif, Carlsbad, CA). The beads were collected by centrifugation and washed once with ChIP buffer 1 and thrice with ChIP buffer 2 (Active Motif). ChIP DNA was eluted in 100 μl of Elution Buffer AM2 (Active motif) and reverse cross-linked at 65°C for 6 h. The DNA samples were eluted in 30 μl of elution buffer using a MiniElute kit (Qiagen, Hilden, Germany). Samples were analyzed by qPCR (**Table 2**).

### RNA-seq and data analysis

Total RNA from the iWAT of P3 and P20 mice was isolated using the RNeasy Lipid Tissue Mini kit (Qiagen) following the kit instructions, and sequencing was performed using Novogene sequencing service. Briefly, the integrity and quantity of the RNA were assessed using Agilent 2100. Strand-specific libraries were generated using the TruSeq Stranded Total RNA Library Prep Kit (Illumina). cDNA libraries were paired-end sequenced (6 Gb) on a sequencing platform and strategy NovaSeq PE150. Raw data were processed through fastp software. In this step, clean reads were obtained by removing adapters, poly-N, and low-quality reads from raw data. At the same time, Q20, Q30, and GC content were calculated. Reads were aligned to the mouse genome (NCB137/mm10). The index of the reference genome was built using Hisat2 v2.0.5 and paired-end clean reads were aligned to the reference genome using Hisat2 v2.0.5. Feature counts v1.5.0-p3 were used to count the read numbers mapped to each gene. The fragments per kilobase of transcript sequence per millions (FPKM) of each gene were calculated based on the gene length and reads count mapped to this gene. Differential expression analysis of two groups was performed using the DESeq2 R package (1.20.0), and the resulting *p*-values were adjusted using Benjamini and Hochberg’s approach for controlling the false discovery rate. A significance cutoff of FDR ≤ 0.05 was applied. Downregulated and upregulated genes were distinguished through fold change analysis. Heat maps were generated by Gene Cluster 3.0 and TrueView 3.0 alpha 3.0 using log2 mean-normalized values.

### ChIP-Seq analysis

Frozen iWAT (note: three iWAT samples pooled into one sample) was sent to Active Motif Services (Carlsbad, CA) for ChIP-Seq. Active Motif prepared chromatin, performed ChIP reactions, generated libraries, sequenced the libraries, and performed basic data analysis. In brief, iWAT was submersed in PBS + 1% formaldehyde and incubated at room temperature for 15 min. Fixation was stopped by the addition of 0.125 M glycine. The tissue pieces were then treated with a TissueTearer, spun down, and washed 2× in PBS. Chromatin was isolated by adding a lysis buffer and disrupted with a Dounce homogenizer. Lysates were sonicated, and the DNA was sheared to an average length of 300–500 bp with Active Motif’s EpiShear probe sonicator (cat# 53051). Genomic DNA (Input) was prepared by treating aliquots of chromatin with RNase, proteinase K, and heat for de-cross-linking, followed by SPRI bead clean up (Beckman Coulter) and quantitation by Clariostar (BMG Labtech). Extrapolation to the original chromatin volume allowed the determination of the total chromatin yield. An aliquot of chromatin (30 μg) was precleared with protein A agarose beads (Invitrogen). Genomic DNA regions of interest were isolated using 4 μg of antibody against H3K27Ac (Active Motif, cat# 39133, lot# 06921014). Complexes were washed, eluted from the beads with SDS buffer, and subjected to RNase and proteinase K treatment. Cross-links were reversed by incubation overnight at 65°C, and ChIP DNA was purified by phenol-chloroform extraction and ethanol precipitation. Quantitative PCR (QPCR) reactions were carried out in triplicate on specific genomic regions using SYBR Green Supermix (Bio-Rad). The resulting signals were normalized for primer efficiency by carrying out QPCR for each primer pair using Input DNA. Illumina sequencing libraries (a custom type, using the same paired read adapter oligonucleotides described by 29) were prepared from the ChIP and Input DNAs on an automated system (Apollo 342, Wafergen Biosystems/Takara). After a final PCR amplification step, the resulting DNA libraries were quantified and sequenced on Illumina’s NextSeq 500 (75-nt reads, single end). Reads were aligned to the mouse genome (mm10) using the BWA algorithm (default settings). Duplicate reads were removed, and only uniquely mapped reads (mapping quality ≥ 25) were used for further analysis. Alignments were extended *in silico* at their 3′-ends to a length of 200 bp, the average genomic fragment length in the size-selected library, and assigned to 32-nt bins along the genome. The resulting histograms (genomic “signal maps”) were stored in bigwig files. Peak locations were determined using the MACS algorithm (30) (v2.1.0) with a *p*-value cutoff of = 1e-7. Peaks that were on the ENCODE blacklist of known false ChIP-Seq peaks were removed. To account for the different sequencing depths between samples, the BAM files generated from MACS were RPKM normalized to sequencing depth using the bam Coverage function in the Galaxy Deep tool, and the bigwig files were generated accordingly (31. The relative intensity of each H3K27Ac signal is further calculated via the compute Matrix function with the RPKM normalized bigwig files and bed files from the peak calling as inputs by calculating the area under the curve.

### Gene ontology analysis

DAVID (Version 6.8) (32) (https://david.ncifcrf.gov/home.jsp) and GREAT (Version 3.0.0) (33) were used to perform Gene Ontology analysis. Briefly, gene names were first converted to DAVID-recognizable IDs using the Gene Accession Conversion Tool. The updated gene list was then subjected to GO analysis using all *Mus musculus* genes as background and with the Functional Annotation Chart function. GO_BP_DIRECT and/or KEGG-PATHWAY were used as GO categories for analyses. Only GO terms with a *p*-value smaller than 0.05 were included for further analysis. For GREAT analysis, a −500 bp to 500 bp window of TSS for each gene was input as a bed file, and enriched MSigDB pathways were generated. The criteria for associating genomic regions with genes are as follows: each gene is assigned a basal regulatory domain of a minimum distance upstream and downstream of the TSS (regardless of other nearby genes) (proximal 5 kb upstream, 1 kb downstream, plus distal up to 100 kb). The gene regulatory domain is extended in both directions to the nearest gene’s basal domain but no more than the maximum extension in one direction.

### Motif analysis

Motif analysis was performed with the SeqPos motif tool (version 0.590) embedded in Galaxy Cistrome using all motifs within mouse reference genome mm10 as background. To identify the GABPα binding motif in the H3K27ac region, the MISP (Motif-based Interval Screener with PSSM) tool embedded in Galaxy Cistrome was used. GABPα motif CTTCC (motif ID MC00367) was used as input.

### Statistical analysis

All the data are presented as means ± SEM. Statistical analysis was performed using GraphPad Prism version 9.0.0 (San Diego, CA). The statistical difference between the two groups was determined by using a two-tailed Student’s *t*-test. One-way analysis of variance followed by the Tukey multiple-comparisons test was applied to compare more than two groups. The differences between the groups were considered statistically significant at *p* ≤ 0.05. *p*-values for the figures are indicated in the corresponding figure legends. *In vitro* experiments were performed at least two times in triplicate.

## Results

### Peri-weaning iWAT has elevated acetylated H3K27 levels

**Figure 1:**
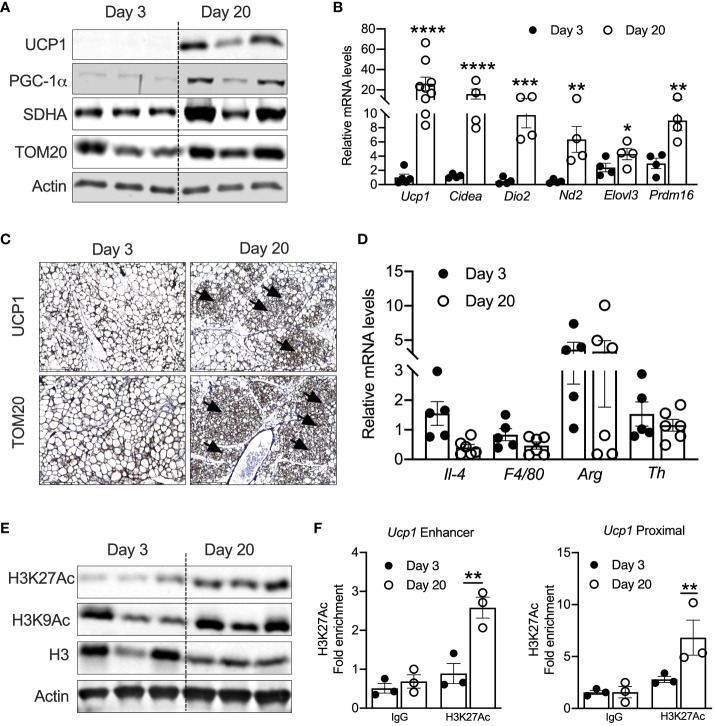
**(A)** Western blot showing beige markers in iWAT of P20 and P3 mice. **(B)** Relative mRNA expression of thermogenic adipocyte markers in iWAT of P20 and P3 mice. **(C)** Immunohistology images showing UCP1 and TOM20 in iWAT of P3 and P20 mice. Magnification 20× (*n* = 3–4 mice) **(D)** Relative mRNA levels showing M1 and M2 macrophage markers in iWAT. **(E)** Western blot for markers showing acetylated histone H3K27 in iWAT. **(F)** H3K27ac Chip-qPCR analysis for *Ucp1 enhancer* and *Ucp1 proximal* in iWAT from P20 and P3 mice. All the data are presented as mean ± SEM. *p < 0.05, **p < 0.01, ***p < 0.001, or ****p < 0.0001 as analyzed by one-way ANOVA (Tukey multiple-comparisons test) and two-tailed Student’s *t*-test.

Previous studies have shown that UCP1-positive beige adipocytes appear in iWAT at P20 (17, 18). Consistently, iWAT of P20 mice showed increased mRNA levels of thermogenic genes such as *Ucp1*, cell death-inducing DFFA-like effector A (*Cidea*), *Dio2, Nd2, Elovl3*, and *Prdm16.* Similarly, protein levels of UCP1, PGC-1α, and mitochondrial-related proteins such as SDHA and TOM20 were elevated in the iWAT of P20 compared to P3 mice (**Figures 1A, B**). Immunohistochemical analysis in the iWAT of P20 mice independently confirmed the presence of multilocular beige adipocytes and higher expression of UCP1 and TOM20 (**Figure 1C**). Next, we interrogated the mechanism that induces neonatal beige adipogenesis. Eosinophils and type 2 cytokines promote beige adipogenesis by activating M2 macrophages expressing tyrosine hydroxylase (*Th*) (34, 35). The catecholamine released from M2 macrophages induces beige adipogenesis in iWAT (36). However, we found no difference in the mRNA levels of *Th*, *Il-4, Arg1*, and *F4/80* between the iWAT of P3 and P20 mice (**Figure 1D**), suggesting that neonatal beige adipogenesis is not driven by sympathetic nerve innervation and type 2 immune signaling.

Peri-weaning is an important transition period in development, where critical physiological pathways are tightly regulated by epigenetic mechanisms (37). Recent studies implicate that epigenetic mechanisms control the transcription of beige-associated genes in adult mice (16, 38). Therefore, we assessed the epigenetic changes in the iWAT of P20 mice, which revealed increased levels of acetylated histone 3 at lysine 9 and 27 (H3K9ac and H3K27ac) (**Figure 1E**). H3K9ac and H3K27ac are the major epigenetic histone modifications that denote active gene transcription (24). Recent studies have shown that transcriptional changes during beige fat development from white fat are associated with epigenetic landscape changes, particularly histone acetylation of H3K27 at beige-specific enhancers but not at white-specific enhancers (16, 39). Therefore, we performed H3K27ac ChIP analysis in the neonatal iWAT to assess whether UCP1 is epigenetically activated. The iWAT of P20 mice showed a significant increase in H3K27ac enrichment on both the enhancer and proximal promoter regions of *Ucp1* gene (**Figure 1F**), suggesting that epigenetic mechanisms potentially induce UCP1, the *bona fide* marker of beige adipocytes (25).

### Peri-weaning beige fat is associated with changes in the epigenetic landscape

**Figure 2:**
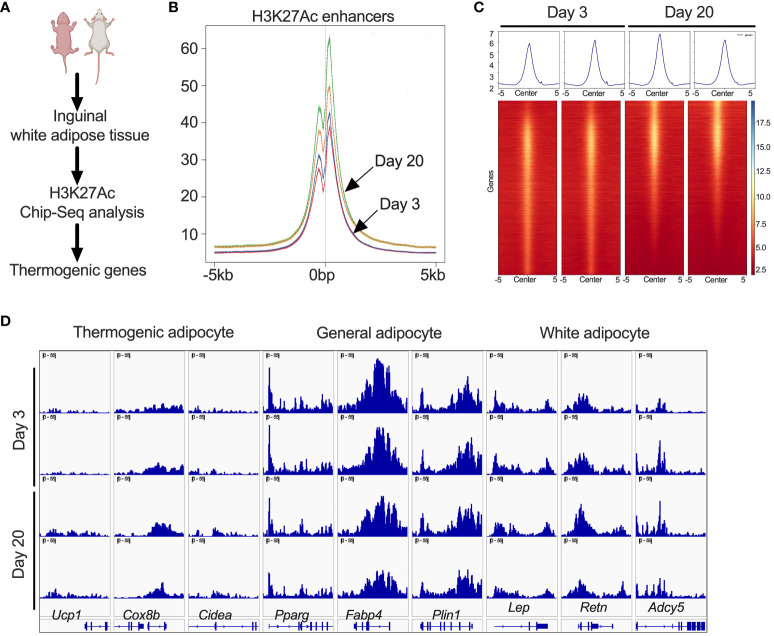
**(A)** Schematic showing H3K27ac ChIP-seq in iWAT of P20 and P3 mice. **(B)** Distribution plot for H3K27ac poised enhancers. **(C)** Heat map of H3K27ac poised enhancers in iWAT that are stronger in P20 mice than in P3 mice. **(D)** H3K27ac ChIP-seq data centered on the TSS (±5 kb) of thermogenic, general, and white adipocyte marker genes.

Since iWAT of P20 mice showed increased H3K27ac, we performed ChIP-seq analysis to define the promoter and enhancer regions that are epigenetically active during the peri-weaning beige adipogenesis (**Figure 2A**). Our analysis revealed unique hyper- and hypo-acetylated H3K27 peaks in the iWAT of P20 compared to P3 mice, suggestive of epigenetically activated and repressed genes, respectively (**Figure 2B**). We then annotated the hyper- and hypoacetylated sites using a window of ±10 kb from the transcription start sites (**Figure 2C**). This analysis revealed chromatin regions of beige-associated genes, such as *Ucp1*, *Cidea*, and *Cox8b* with higher H3K27 acetylation peaks in P20 compared to P3 mice (**Figure 2D**). However, we found no difference in H3K27ac marks on the chromatin regions of general adipose-specific genes such as *Pparγ*, *Fabp4*, and *Plin* and white adipocyte-specific markers such as *Lep*, *Retn*, and *Adcy5* (**Figure 2D**). This suggests that epigenetic mechanisms differentially regulate white and beige genes in neonatal iWAT.

### Peri-weaning iWAT displays beige-specific transcriptional profile changes

**Figure 3:**
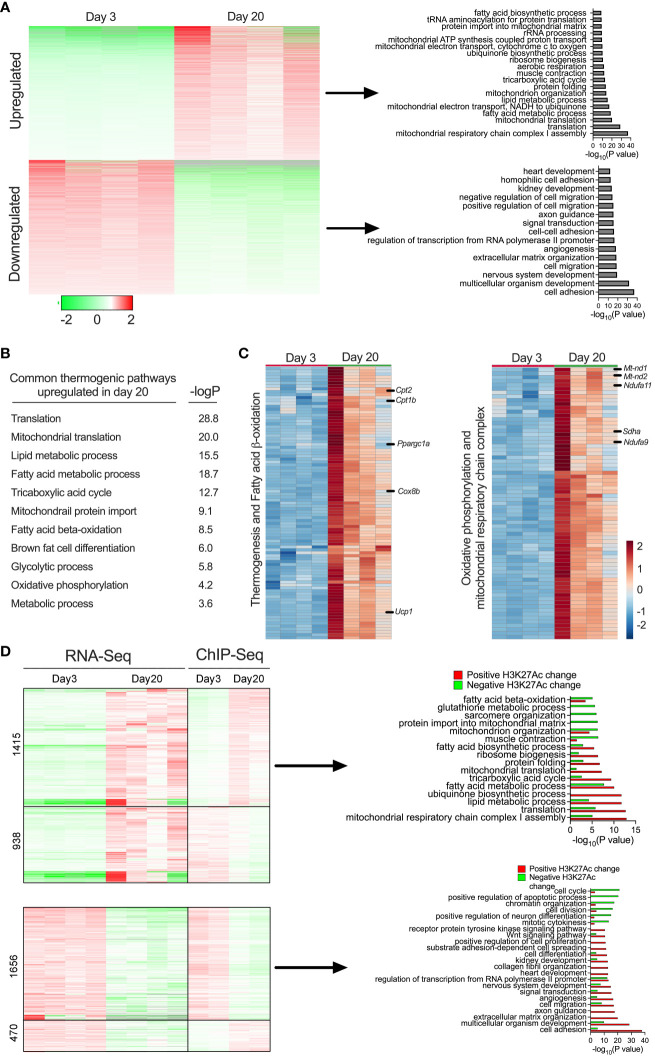
**(A)** Heat map showing differentially expressed gene profiles and GO pathway analysis of gene clusters upregulated and downregulated in P20 and P3 mice. **(B)** GO enrichment pathway analysis showing thermogenic genes and corresponding *p*-values of the upregulated genes iWAT of P20 mice. **(C)** Heat map of upregulated genes in the GO category of thermogenesis, fatty acid oxidation, oxidative phosphorylation, and mitochondrial metabolism (*n* = 4 per group) **(D)** Heat map of gene expression profiles of RNA-seq and ChIP-seq and GO pathways of up- or downregulated genes with positively or negatively associated H3K27ac changes in iWAT of P20 compared to P3 mice.

We then performed RNA-seq analysis in the iWAT of P3 and P20 mice to define the transcriptional profile. Principal component analysis (PCA) of the RNA-Seq data showed that iWAT from P20 exhibited a distinct molecular signature from iWAT of P3, which explains 30.3% of the variance (**Supplementary Figure 1A**). Notably, 2,604 genes were downregulated and 3,101 genes were upregulated in the iWAT of P20 mice (**Supplementary Figure 1B**). We then sought to understand the biological pathways that might be affected in the iWAT of P20 compared to P3 mice. Gene ontology (GO) enrichment analysis revealed that lipid metabolism pathways, including the fatty acid metabolic process, fatty acid biosynthesis process, fatty acid beta-oxidation, and carbohydrate metabolic process were upregulated. Mitochondrial metabolism and function-related pathways, including mitochondrial translation, mitochondrial electron transport chain, mitochondrial organization, mitochondrial ATP synthesis, and tricarboxylic acid cycle, were specifically upregulated in P20 mice. In addition, DNA repair pathway genes related to protein transport to mitochondria and translation were induced in P20 mice. In contrast, the common downregulated genes were implicated in cell adhesion, multicellular organism development, nervous system development, cell migration, and angiogenesis (**Figure 3A**). These results indicate that iWAT adipocytes undergo a profound shift in their transcriptome from P20 and P3 mice.

Beige cells are characterized by high levels of thermogenic capacity, mitochondrial metabolism, and fatty acid β-oxidation (5, 25) (**Figure 3B**). Our in-depth analysis showed that 124 genes involved in thermogenesis were significantly increased in the iWAT of P20 mice (**Figure 3C**). For instance, the most well-known thermogenic gene, *Ucp1*, and genes involved in fatty acid β-oxidation (*Cpt1b* and *Cpt2*) and mitochondrial biogenesis-related genes, such as *Pgc1α, Sdha, Sdhb, Nd2, Nd1*, and *Nd4*, were among the top upregulated genes. Thermogenesis occurs through the activity and increased expression of several genes in inner mitochondrial membrane that uncouple substrate oxidation from ATP synthesis to generate heat (40). We observed that genes involved in coupled respiration via ATP synthases, such as *Atp50, Atp5b, Atp5h, Atp5g2*, and *Atp5d*, were significantly increased. Furthermore, a set of genes encoding for NADH dehydrogenase 1 alpha subcomplex, such as *Ndufa9, Ndufaf3, Ndufa2, Ndufa7, Ndufa4, Ndufa6*, and *Ndufb10*, were significantly increased. These proteins belong to a subunit of NADH:ubiquinone oxidoreductase, located in the mitochondrial inner membrane with a critical role in the electron transport chain (41) (**Figure 3C**). The data suggest that molecular characteristics of the peri-weaning beige adipocytes are similar to canonical beige adipocytes.

We further integrated RNA-seq and ChIP-seq data to identify the pathways that were potentially driven by epigenetic mechanisms. This analysis identified several *bona fide* thermogenic pathways upregulated or downregulated in P20 mice compared to P3 mice. Among them, genes involved in beige fat development, such as mitochondrial respiratory chain complex 1 assembly, lipid metabolic process, fatty acid metabolic process, mitochondrial translation, and fatty acid biosynthesis process, were upregulated and positively associated with H3K27ac enrichment at their gene regulatory regions. In contrast, genes involved in cell adhesion, multicellular organism development, angiogenesis, and cell migration were downregulated and associated with decreased H3K27ac levels in the iWAT of P20 mice (**Figure 3D**). Thus, the combination of the transcriptomic and epigenetic approaches identified the markers/signatures of beige fat that are epigenetically regulated in the neonatal iWAT.

### GABPα as a regulator of peri-weaning beige adipogenesis

**Figure 4:**
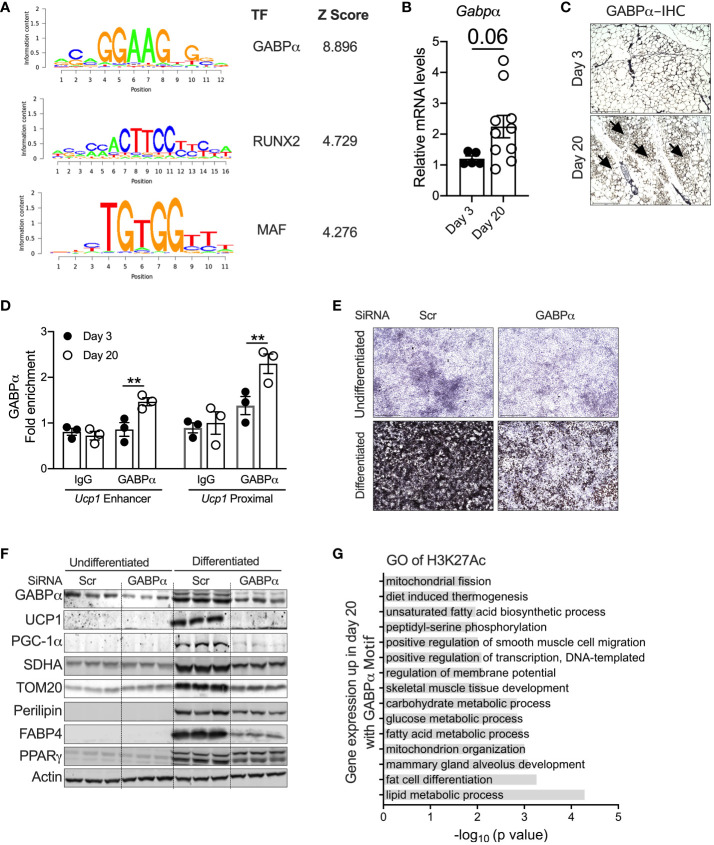
**(A)** Position weight matrix logos of beige-enriched motifs and ranks of each motif are represented by *Z*-score. **(B)** Relative mRNA levels of Gabpα in iWAT of P20 and P3 mice. **(C)** Immunohistology showing GABPα in iWAT. Magnification 20× (*n* = 2–3 mice). **(D)** Chip-qPCR analysis for *Ucp1 enhancer* and *Ucp1 proximal* for GABPα in iWAT from P20 and P3 mice. **(E)** Oil Red O staining of the SVF cell line transfected with siRNA against *Gabpα* or scramble and differentiated into beige adipocytes for 6 days (magnification 4×). **(F)** Western blot showing beige adipocyte markers in the inguinal SVF cell line transfected with siRNA against *Gabpα* or scramble and differentiated into beige adipocytes. **(G)** GO pathway of H3K27ac with potential GABPα motif upregulated in P20 mice. All the data are presented as mean ± SEM. **p < 0.01 as analyzed by one-way ANOVA (Tukey multiple-comparisons test) and two-tailed Student’s *t*-test.

To identify the potential transcriptional pathways/circuits that control peri-weaning beige adipocyte development and differentiation, we performed motif analysis on all H3K27Ac peaks that show at least a fourfold increase in P20 compared to P3 (*p* < 0.05). Motif analysis of the hyperacetylated H3K27 region identified several ETS family of transcription factors, particularly DNA-binding motifs for ETS1, ETV1, ETV7, Fli1, ERG, GA-binding protein-α (GABPα), RUNX2, and MAF, all of which were significantly enriched in P20 iWAT mice (**Figure 4A**). The *Z*-scoring among these factors showed a hyperrepresentation of the GABPα binding motif in the active chromatin regions of neonatal iWAT. To further elucidate whether GABPα is linked to neonatal beige adipogenesis, we assessed the expression of GABPα in iWAT, which revealed elevated mRNA in iWAT of P20 mice (**Figure 4B**). Moreover, immunohistochemical analysis showed increased expression of GABPα in multilocular adipocytes in iWAT of P20 mice (**Figure 4C**). We then performed GABPα ChIP analysis in the iWAT of P20 mice, which showed a significant increase in GABPα chromatin enrichment on the *Ucp1* promoter (**Figure 4D**). To understand whether GABPα is required for beige adipocyte differentiation, we knocked down GABPα in the mouse inguinal SVF cell line using siRNA and then differentiated it into beige adipocytes. Knockdown of *Gabpa* significantly attenuated beige adipogenesis (**Figure 4E**), as revealed by decreased expression of beige-specific markers such as UCP1, and other beige-associated genes such as PGC-1a, SDHA, and TOM20. However, expression of general adipogenesis markers such as PERILIPIN, FABP4, and PPARγ was unchanged (**Figure 4F**). Collectively, we found that epigenetically active chromatin regions in neonatal iWAT are enriched with binding sites of GABPα, which is crucial for beige adipocyte differentiation.

To further identify the transcriptional pathways regulated by GABPα, we performed GO analysis on the specific sets of H3K27Ac peaks harboring strong GABPα binding motifs. We observed that several pathways involved in fat cell differentiation, thermogenesis, and lipid metabolic processes, including fatty acid biosynthesis and carbohydrate metabolism, mitochondrial metabolism, and glucose metabolism, were highly upregulated (**Figure 4G**). Taken together, these results suggest that the GABPα binding sites have H3K27ac peaks that were induced and involved in beige adipogenesis.

### Peri-weaning beige adipocytes are glycolytic in nature

**Figure 5:**
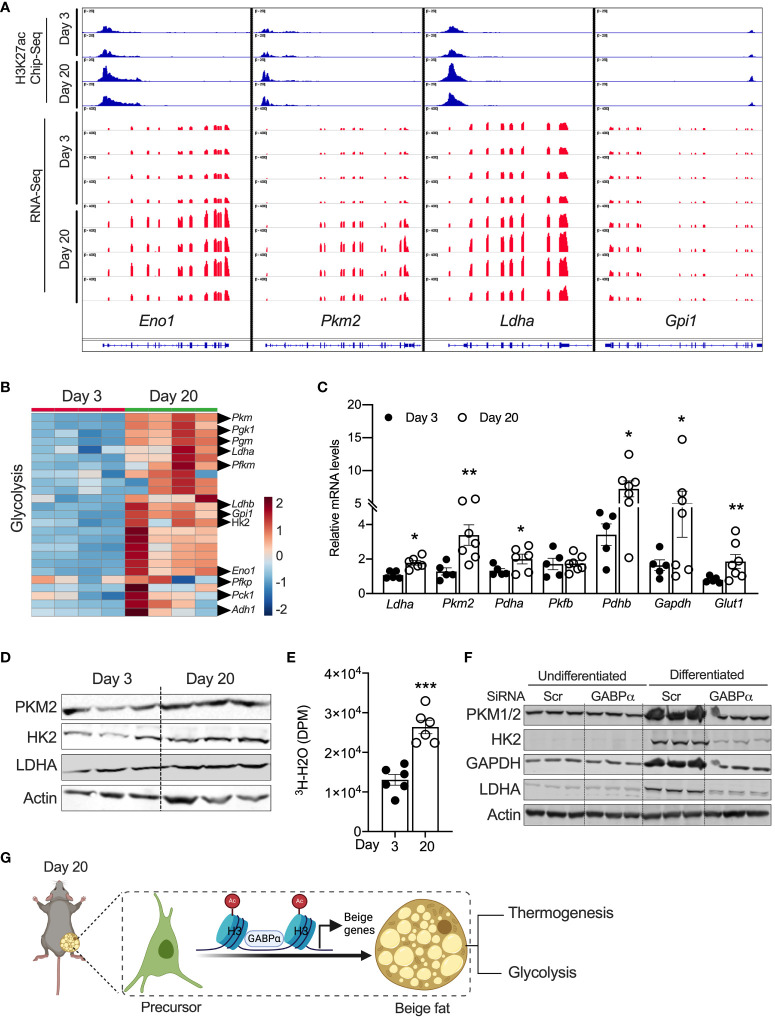
**(A)** Distribution of H3K27ac poised enhancers in glycolysis-related genes in iWAT of P20 and P3 mice. **(B)** Heat map of upregulated genes in the “glycolysis” GO category in iWAT of P20 and P3 mice. **(C)** Relative mRNA expression for glycolysis-related genes in iWAT. **(D)** Western blot showing proteins for glycolysis in iWAT. **(E)** *Ex vivo* glycolysis analysis in iWAT of P20 and P3 mice. **(F)** Western blot showing glycolysis-related proteins in the inguinal SVF cell line transfected with siRNA against *Gabpα* and differentiated into beige adipocytes. **(G)** Schematic showing the mechanism of beige adipocyte induction in iWAT of P20 mice. The Schematic figure was generated using BioRender. All the data are presented as mean ± SEM. *p < 0.05, **p < 0.01, or ***p < 0.001 as analyzed by two-tailed Student’s *t*-test.

Chen et al. characterized a new class of beige adipocytes known as glycolytic beige adipocytes that control glucose homeostasis and thermogenesis (26). Notably, these glycolytic beige adipocytes are regulated by GABPα. We assessed the expression signatures of metabolic genes in neonatal iWAT. Our GO enrichment analysis of RNA-Seq data show that several genes involved in glycolysis, carbohydrate metabolism, and carbon metabolism were enriched in P20 iWAT relative to P3 mice. For example, expression of glycolytic genes, including *Eno1*, *Ldha, Pck1, Hk2*, *Gpi1*, and *Pkm2*, was significantly higher in P20 iWAT relative to P3 (**Figures 5A, B**). ChIP-seq analysis in iWAT of P20 mice had strong H3K27ac peak signals in the chromatin regions of *Eno1, Pkm2, Ldha*, and *Gpi1* (**Figures 5A, B**). Consistently, qRT-PCR and Western blot analysis showed upregulation of the mRNA and protein of glycolytic genes in the iWAT of P20 mice, compared to P3 mice (**Figures 5C, D**), suggesting that neonatal iWAT possesses signatures of glycolytic beige. This led to the hypothesis that neonatal iWAT may actively utilize glucose through enhanced glycolysis, and therefore, we assessed the glycolytic rates in the iWAT of P20 mice *ex vivo* using 2-deoxyglucose. We found that iWAT from P20 mice exhibited significantly higher rates of glycolysis than P3 mice (**Figure 5E**). Furthermore, the knockdown of *Gabpα* in inguinal SVF cells significantly reduced the expression of glycolytic proteins such as PKM2, HK2, GAPDH, and LDHA in differentiated beige adipocytes (**Figure 5F**). These results indicate that GABPα is involved in inducing neonatal beige fat with enhanced glucose metabolism, i.e., glycolytic beige fat.

## Discussion

Beige adipocytes alter systemic energy metabolism and protect against several metabolic diseases (1, 5). Recent studies show that beige adipocytes are found in neonates and are crucial for the sympathetic innervation of iWAT (17, 20). However, the mechanisms underlying their regulation remain elusive. We report that the development of neonatal beige adipocytes is associated with epigenetic changes, particularly H3K27ac at beige-specific enhancers and promoters. The integration of ChIP-seq and RNA-seq analysis revealed elevated H3K27ac peaks on beige fat-specific enhancers without affecting the white adipose-specific enhancers. Furthermore, motif enrichment analysis on H3K27ac peaks identified GABPα as a critical transcriptional regulator essential for beige adipogenesis. Finally, we uncovered that these beige adipocytes are glycolytic in nature (**Figure 5G**). Thus, our data show that epigenetic mechanisms drive peri-weaning development of beige adipocytes via GABPα.

Adipose tissue plasticity or remodeling, including white adipose tissue browning, is determined by factors that facilitate changes in gene expression, leading to altered cell identity (3, 42). Recent studies indicate that temperature changes maintain a stable chromatin state and alter adipocyte morphology and function (16). For example, beige adipocytes convert to white adipocytes during cold stimuli withdrawal but retain an epigenomic memory defined by an array of poised enhancers that help to activate a thermogenic program upon cold re-exposure (16). This indicates that beige adipocytes rapidly alter their morphology and gene expression, but the chromatin state changes in response to external cues. Additionally, nucleosome binding proteins, such as high-mobility group N (HMGN), modulate white adipose tissue browning by epigenetically stabilizing the white adipocyte cell identity (43). Similarly, BCL6 regulates beige adipogenesis by altering the chromatin state, and this was achieved by simultaneously activating beige-specific enhancers while suppressing white-specific markers (44). Thus, the induction of beige adipogenesis and epigenetic changes was well established in adult mice; however, at what stage of development these epigenetic marks were formed is unknown.

The peri-weaning is an important transition point where several physiological systems undergo remodeling and maturation to adapt from mother’s milk to solid food (37, 45). Several paracrine and endocrine signals converge during this period to regulate histone modification, nucleosome organization, chromatin topology, and dynamics (45). For example, fibroblast growth factor 21 (FGF21) and ketone bodies act as epigenetic modifiers (37, 46, 47). Furthermore, a recent study showed that transcription factor B-cell leukemia/lymphoma 6 (BCL6) is required for peri-weaning iWAT remodeling into beige adipocytes. Since BCL6 regulates adipose tissue browning in adult mice through epigenetic mechanisms (44), we asked whether epigenetic changes play a role in peri-weaning beige adipogenesis. Our H3K27ac ChIP-seq and RNA-seq integration analysis revealed elevated H3K27ac levels in beige fat-specific enhancers in iWAT but not white fat-specific enhancers. Moreover, ChIP-seq and RNA-seq integration showed enrichment of pathways involved in thermogenesis, mitochondrial metabolism, and fatty acid oxidation. Together, these data suggest that peri-weaning beige fat development is driven by epigenetic mechanisms.

Our motif enrichment analysis for H3K27ac peaks identified a DNA-binding motif for GABPα, which was significantly increased in peri-weaning iWAT. GABPα belongs to E26 transformation-specific (ETS) factors that heterodimerize with GABPβ subunit to form a functional GABP transcription factor complex, also known as nuclear respiratory factor 2 (NRF-2) or adenovirus E4 transcription factor 1 (E4TF-1) (48). Earlier studies showed that GABPα regulates lineage-restricted myeloid and lymphoid genes involved in innate immunity, cell cycle control in fibroblasts, and several genes necessary for mitochondrial metabolism (49–51). Moreover, genetic disruption and knockdown of mouse *Gabpa* resulted in embryonic lethality and mitochondrial dysfunction, including reduced mitochondrial mass, ATP production, oxygen consumption, and mitochondrial proteins (52, 53). *Gabpa* was shown to regulate mitochondrial biogenesis and OXPHOS program by interacting with PGC1α (54). A recent study showed that GABPα drives the development of glycolytic beige fat independent of β-AR signaling (26). Thus, we tested whether GABPα regulate peri-weaning beige adipogenesis. Three findings from our study supported our speculation; first, *Gabpa* levels increase in the iWAT of P20 mice. Second, H3K27ac Chip-qPCR analysis in iWAT of P20 mice showed GABPα binding regions on beige-specific enhancers. Third, bioinformatic analysis revealed the upregulation of GABPα binding sites associated with H3K27ac peaks on beige genes in P20 mice. The binding of ETS transcription factors, including GABP, depends on the poised or active chromatin state of enhancers and promoters (53). A strong association between H3K27Ac peaks and GABPα binding sites in beige genes suggests that epigenetic events preceded GABP-mediated regulation of beige adipogenesis. Therefore, investigating the upstream mechanisms that regulate the epigenetic activation of GABPα will help us harness the therapeutic potential of beige adipocytes in metabolic diseases.

Beige adipocytes induced by the β-AR signaling regulate systemic energy metabolism through UCP1-dependent and independent mechanisms, such as anti-inflammatory and anti-fibrosis (55, 56). A recent study identified a new class of β-AR-independent stimulation of beige adipocytes that control thermogenesis and glucose homeostasis (26). Thus, multiple sub-types of beige adipocytes (canonical and glycolytic beige) with distinct biological functions are induced by various external stimuli. Unlike adult mice, peri-weaning beige adipocytes develop under physiological conditions, independent of β-AR signaling and temperature (17). We found that the peri-weaning beige adipocytes are characterized by an increased thermogenic program, including mitochondrial metabolism, oxidative phosphorylation, lipid metabolic processes, and fatty acid oxidation mechanisms. Additionally, we found that these beige adipocytes are highly enriched with glycolytic genes and functionally oxidize glucose. Thus, our data suggest that peri-weaning beige adipocytes are glycolytic beige adipocytes, supported by a strong positive association between GABPα expression and glycolytic gene expression in the peri-weaning beige adipocytes.

In summary, we report that the development of peri-weaning beige adipocytes is associated with elevated H3K27ac levels on beige adipocyte-specific enhancers. H3K27ac ChIP-seq and RNA-seq displayed strong signals on thermogenic genes but not white adipocyte markers and identified GABPα as a critical transcriptional driver associated with neonatal beige adipogenesis. Gene signature denotes that the neonatal beige adipocytes are thermogenic and glycolytic. A better understanding of neonatal beige adipocyte heterogeneity may provide new insights into the molecular basis of adaptation or development in physiology and pathological conditions.

## Data availability statement

The datasets presented in this study can be found in online repositories. The names of the repository/repositories and accession number(s) can be found below: https://dataverse.harvard.edu/dataset.xhtml?persistentId=doi:10.7910/DVN/S5TC2V, 10.7910/DVN/S5TC2V.

## Ethics statement

The animal study was approved by Institutional Animal Care and Use Committee (IACUC) at the University of Pittsburgh. The study was conducted in accordance with the local legislation and institutional requirements.

## Author contributions

RM: Conceptualization, Formal analysis, Investigation, Methodology, Validation, Visualization, Writing – original draft, Writing – review & editing. BZ: Data curation, Formal analysis, Investigation, Methodology, Writing – original draft, Writing – review & editing. SK: Data curation, Formal analysis, Investigation, Methodology, Writing – original draft, Writing – review & editing. VN: Writing – original draft, Writing – review & editing, Methodology, Formal analysis. KM: Writing – original draft, Writing – review & editing, Methodology, Formal analysis. MJ: Formal analysis, Methodology, Writing – original draft, Writing – review & editing. SR: Conceptualization, Funding acquisition, Methodology, Resources, Supervision, Writing – original draft, Writing – review & editing.

## Supplementary material

Supplementary Figure 1
**(A)** PCA of transcriptome from iWAT of P20 and P3 mice. Each dot indicates an individual mouse. **(B)** Volcano plot showing up- and downregulated genes in iWAT of P20 and P3 mice and adjusted *p*-value < 0.05.
