# Supplementary figures and images for: Epigenetically active chromatin in neonatal iWAT reveals GABPα as a potential regulator of beige adipogenesis
Source: Front Endocrinol (Lausanne). 2024 May 3;15:1385811. doi: 10.3389/fendo.2024.1385811 (PMC11099907; doi:10.3389/fendo.2024.1385811)

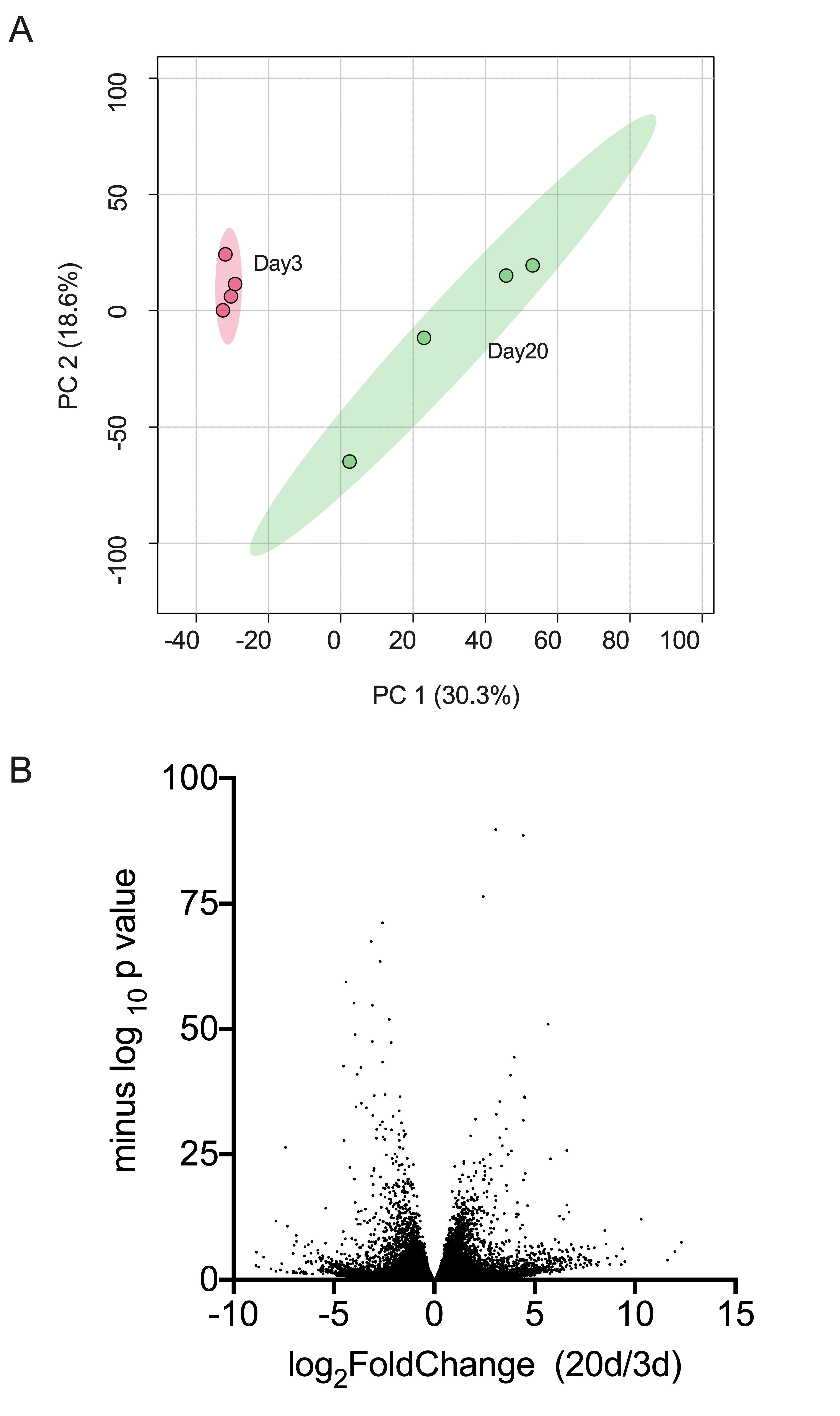

Supplement: Supplementary Figure 1 — (A) PCA of transcriptome from iWAT of P20 and P3 mice. Each dot indicates an individual mouse. (B) Volcano plot showing up- and downregulated genes in iWAT of P20 and P3 mice and adjusted p-value < 0.05. [file Image_1.jpg]
